# Fibroblast growth factor 3 promotes spontaneous mammary tumorigenesis in Tientsin albino 2 mice *via* the FGF3/FGFR1/STAT3 pathway

**DOI:** 10.3389/fonc.2023.1161410

**Published:** 2023-07-11

**Authors:** Lankai Chen, Xipeng Zhang, Guisheng Liu, Shuo Chen, Minying Zheng, Siwei Zhu, Shiwu Zhang

**Affiliations:** ^1^Nankai University School of Medicine, Nankai University, Tianjin, China; ^2^Department of Colorectal Surgery, Tianjin Union Medical Center, Tianjin, China; ^3^Department of Pathology, Tianjin Union Medical Center, Tianjin, China

**Keywords:** mouse mammary tumor virus, fibroblast growth factor 3, spontaneous breast cancer, Tientsin albino 2, MA-891

## Abstract

**Introduction:**

Tientsin albino 2 (TA2) mice can develop spontaneous breast cancer (SBC), which is associated with multiple pregnancies and infection with the mouse mammary tumor virus (MMTV). In this study, we sought to elucidate the molecular mechanisms underlying the development of SBC in TA2 mice induced by MMTV.

**Methods:**

The integration site of MMTV in TA2 SBC was identified using whole-genome sequencing. The expression of fibroblast growth factor 3 (FGF3) in SBCs and normal breast tissues was compared. The primary cell line, TA-1106, derived from SBC, was cultured. The proliferation, cell cycle, migration, invasion, and tumorigenicity abilities, as well as the expression of epithelial-mesenchymal transition-related proteins, phosphorylated STAT3, and phosphorylated Akt, were assessed in MA-891cell line from TA2 and TA-1106 cells after FGF3 knockdown. The binding of FGF3 to FGF receptor 1 (FGFR1) was determined by co-immunoprecipitation. Additionally, the relationship between STAT3 and Akt phosphorylation was investigated using a small molecule inhibitor and STAT3 knockdown.

**Results:**

MMTV integrated upstream of the FGF3 gene, and the FGF3 protein was highly expressed in TA2 SBCs. FGF3 knockdown in MA-891 and TA-1106 decreased their proliferation, migration, and invasion abilities, affected the cell cycle and expression of epithelial-mesenchymal transition-related proteins, and inhibited the growth of animal xenografts. FGF3 binds to FGFR1, and either FGF3 or FGFR1 knockdown decreases STAT3 and Akt phosphorylation levels. Inhibition of phosphorylation or expression of STAT3 resulted in decreased Akt phosphorylation levels. Inhibition of Akt phosphorylation also resulted in decreased STAT3 phosphorylation levels. Furthermore, treatment of MA-891 and TA-1106 cells with Wortmannin or Stattic caused FGFR1 upregulation in addition to inhibiting Akt or STAT3 phosphorylation.

**Conclusion:**

The results of this study demonstrate that FGF3 plays a significant role in the development of SBC through the FGF3/FGFR1/STAT3 signaling pathway. There is a reciprocal activation between STAT3 and Akt. Inhibition of STAT3 or Akt phosphorylation promoted the expression of FGFR1. Validating the conclusions obtained in this study in human breast cancer (HBC) may contribute to targeted therapy and it is worth exploring whether the homologous sequences of MMTV in HBC have a similar oncogenic effect.

## Introduction

According to 2020 global cancer statistics, breast cancer accounts for 11.7% of all new cancer cases and is the leading cause of cancer-related deaths in females, accounting for 15.5% of all cancer-related deaths in females (1). Pregnancy-associated breast cancer (PABC) refers to breast cancer diagnosed during pregnancy or within one year after delivery and is associated with a poor prognosis. PABC is also more likely to be triple-negative breast cancer (TNBC) (2, 3). Tientsin Albino 2 (TA2) mice are an inbred mouse strain established at Tianjin Medical University. A key feature of TA2 mice is their ability to develop spontaneous breast cancer (SBC) in the absence of external stimuli (4, 5). Around 84.1% of multiparous TA2 mice have SBC, while only 41.4% of virgin TA2 mice do (6). This suggests that estrogen and progesterone may play important roles in the tumorigenesis of SBC. The concentrations of serum 17β-estradiol (E2) and progesterone in TA2 mice increase with the number of pregnancies, and exogenous administration of estradiol and progesterone *via* abdominal injection in ovary-removed virgin TA2 mice results in a higher incidence of SBC (7). In addition, TA2 SBC has been identified as TNBC using pathological analysis (8). Based on these results, the TA2 mouse is an appropriate model for PABC research.

Mouse mammary tumor virus (MMTV) has been shown to be integrated and expressed in TA2 SBC tissues, and virus particles have been observed by electron microscopy (8–10). After the integration of MMTV into the mouse genome, hormones elevated during pregnancy bind to hormone response elements on the MMTV long terminal repeat to activate downstream proto-oncogenes and lead to breast cancer. In 1995, by combining polymerase chain reaction and Southern blot assays, Wang et al. demonstrated that a 660-bp MMTV env-like sequence (MMTVels) with high similarity to the MMTV env gene (95–99%) could be detected in 38.5% (121 out of 314) of unselected human breast cancer (HBC) samples collected from American female breast cancer patients, compared to 6.9% (two out of 29) in breast fibroadenomas and 1.8% (two out of 107) in normal breast tissues (11). Interestingly, MMTVels are more common in PABC than in SBC. This suggests that MMTVels may be related to the tumorigenesis of PABC (12).

*FGF3*, previously known as the *Int-2* gene, is one of the common insertion sites in MMTV. Other common insertion sites include Wnt-1/Int-1 (13), Wnt-3 (14), Wnt-10b (15), int-3/Notch4 (16), int-6 (17), Fgf-4/hst (18), and Fgf-8/AIGF (19). *FGF3* belongs to the *FGF7* subfamily, which is one of five paracrine subfamilies of FGFs (20). Co-activation of the Wingless-related integration site (Wnt) and FGF signaling pathways accelerates mammary tumorigenesis in MMTV-Wnt1 or MMTV-Fgf3 transgenic mice (21). Those with progesterone receptor-negative primary breast cancer who have Int-2/FGF3 amplification have a five-fold greater risk of relapse (22).

In this study, whole-genome sequencing (WGS) was performed on TA2 mice with SBC, and the results confirmed that the virus-host junction was near the *FGF3* gene. The expression levels of FGF3 in SBC and normal breast tissues were compared. TA-1106 (primary culture cells) were obtained from the SBC of TA2. FGF3 plays important roles in proliferation, migration, invasion, and cycle progression *in vitro* and tumorigenesis *in vivo*. Additionally, the mechanism by which FGF3 induces SBC was investigated, and the relationship between STAT3 and Akt was also discussed. In conclusion, the FGF3/FGFR1/STAT3 signaling pathway is one of the mechanisms by which FGF3 promotes cancer. A correlation was observed between STAT3 and Akt activation. Furthermore, the inhibition of STAT3 or Akt activation by small-molecule inhibitors and knockdown of STAT3 by siRNA led to FGFR1 upregulation.

## Materials and methods

### DNA extraction

TA2 mice were purchased from Tianjin Medical University, bred, and allowed to become pregnant until they developed SBC. The average number of pregnancies before developing SBC was approximately four. The TA2 mouse with SBC was sacrificed, and the tumor was stripped for DNA extraction. All animal experiments were performed in accordance with the Guidelines for the Care and Use of Laboratory Animals established by the Chinese Council on Animal Care. Approximately 80 mg of tumor tissue was extracted using sterilized scissors and tweezers, ground in liquid nitrogen, and transferred to an Eppendorf tube. DNA was extracted using the centrifugal adsorption column method, according to the manufacturer’s instructions for the MolPure^®^ Cell/Tissue DNA kit (Yeasen Biotechnology, China).

### Agarose gel electrophoresis and WGS

In brief, a 1% agarose gel was stained with a YeaRed nucleic acid gel stain (Yeasen Biotechnology, China). To each well, 1 µL of DNA sample was added and electrophoresed for 45 minutes at 110 V of constant voltage. Imaging was performed using a ChemiDoc imaging system (Bio-Rad). After the assessment of the DNA samples by agarose gel electrophoresis, WGS of the DNA samples was performed by the Novogene sequencing company (Novogene Co., Ltd., China).

### Protein extraction from the normal breast tissue and SBC

Ten healthy female mice were sacrificed, and their mammary glands were removed. Ten SBC tissues were also collected within a year and stored at -80°C for protein extraction. Remove adipose tissue using a stereomicroscope before extracting proteins from normal breast tissue. Approximately 30 mg of sample was taken from each tissue, and 300 μL of radioimmunoprecipitation assay (RIPA) buffer supplemented with 3 μL PMSF was added. Tissues were lysed on ice for 30 minutes and then centrifuged for 15 minutes at 14,000 rpm and 4°C. The supernatants were transferred to new, pre-cooled Eppendorf tubes. The absorbance of the supernatants at 280 nm (A280) was measured. Supernatants were mixed with 1/4 volume of 5X loading buffer and boiled at 100°C for 10 minutes. Protein samples were stored at -20°C.

### Western blot assay

Briefly, protein samples were loaded onto 10% sodium dodecyl sulfate-polyacrylamide gels and separated by electrophoresis. Subsequently, the separated proteins were electrophoretically transferred onto polyvinylidene fluoride membranes. For 15 minutes, membranes were blocked in QuickBlock™ Blocking Buffer (Beyotime Biotechnology, China). Then, the membranes were incubated with primary antibodies (**Supplementary Table 1**) overnight at 4°C. The next day, membranes were incubated with secondary antibodies for one to two hours. Imaging was performed using a ChemiDoc imaging system (Bio-Rad, USA).

### Primary cell culture

One TA2 mouse with SBC was sacrificed, immersed in 75% alcohol for five minutes, fixed on a sterilized foam board with needles, and then stripped using sterilized surgical blades, scissors, and tweezers. It was washed twice with wash solution (Hanks’ Balanced Salt Solution: 200 U/mL of penicillin and 200 µg/mL of streptomycin) and was cut into 0.5 to 1 mm^3^ pieces in the petri dish with sterilized scissors, and the tissue was moistened with 20% Roswell Park Memorial Institute (RPMI)-1640 complete medium (RPMI-1640 medium: 20% fetal bovine serum [FBS], 100 U/mL of penicillin, and 100 µg/mL of streptomycin). Tissue fragments were transferred to a T25 culture flask coated with rat tail glue and spread using a cell scraper. Two milliliters of 20% RPMI-1640 complete medium was added to the T25 flask, and the T25 flask was then put in the humidified incubator (37°C, 5% CO_2_) for four hours. When adherent tumor cells were observed in the T25 flask (five to seven days after inoculation), the tumor tissue fragments were gently scraped with a sterilized needle, and the tumor cells were digested and inoculated into six-well plates at a low density (approximately 240 cells/well). After two weeks of culture, several cell clones were scraped off with sterilized needles and inoculated into 24-well plates for culturing. When the cell confluence reached approximately 90%, the tumor cells were digested and inoculated into T25 flasks for culturing. The primary cultured SBC cells were named TA-1106.

### Hematoxylin and eosin staining

MA-891 and TA-1106 cells were cultured in six-well plates embedded with sterilized coverslips for one to two days. After washing three times with phosphate-buffered saline (PBS) for three times, the cells were fixed with 75% methanol for 30 minutes. The cells were stained with *hematoxylin* for one minute and *eosin* for one minute. Coverslips were air-dried and sealed with neutral gum.

### Transient siRNA transfection

All siRNA sequences used in this study were synthesized by GenePharma (details of the siRNA sequences are listed in **Supplementary Table 2**). Briefly, cells were seeded in six-well plates at an approximate cell density of 40% the day before transfection and cultured in a 10% RPMI-1640 complete medium. The following day, 5 μL of transfectant (GenePharma, China) and 5 μL (100 pmol) of siRNA were added to 50 μL of the medium and allowed to stand for five minutes. The transfectant and siRNA solutions were then mixed and allowed to stand for 20 minutes. The medium in the six-well plate was replaced with fresh medium containing 10% RPMI-1640 without antibiotics. For the next experiment, the transfectant-siRNA mixture solution was added to each well and incubated at 37°C for 48–72 hours.

### Cell viability cell counting Kit-8 assay

The survival ability of siRNA-NC and siRNA-297 group cells was evaluated using the cell viability cell counting Kit-8 (CCK8) assay. The cells were seeded in 96-well plates at increasing concentrations (1 × 10^3^, 2 × 10^3^, 3 × 10^3^, 4 × 10^3^, and 5 × 10^3^ cells/well) in 100 μL medium with 10% FBS and incubated at 37°C for 48 hours. Then, cells were cultured in 10% RPMI-1640 medium with 10% CCK-8 reagent (Dojndo, Japan) for two hours. Finally, the absorbance of wells at 450 nm was measured using a microplate reader (Bio-Rad, USA).

### Plate clone formation assay

Cell proliferation was evaluated using the plate colony formation assay. Different numbers of cells (60, 120, and 240 cells/well) were cultured in the wells and incubated at 37°C for two weeks. Incubation was terminated when white clumps appeared in the 12-well plates. The cell colonies were fixed with 75% methanol for 30 minutes and then stained with 0.1% crystal violet for 30 minutes at room temperature. The number of clumps in each well was counted to evaluate the proliferative ability of cells in different groups.

### Flow cytometry

Cells transfected with siRNA were trypsinized and centrifuged at 1000 rpm for 5 minutes. The cells were resuspended in 500 µL of PBS and washed twice. A cell cycle kit (BD Biosciences, USA) was then added and allowed to react at room temperature in the dark for 30 minutes. FACSCalibur (BD Biosciences, USA), CellQuest software (BD Biosciences, USA), and Modfit LT software were used for analysis. The forward and side scatter (FSC/SSC) plot was used to observe the overall cells, the FL-2A/FL-2W scatter plot was used to remove adhesion cells, the FL2-H histogram was used to adjust the G1 peak of the cell cycle, and the morphology of the cell cycle peak in the FL-2A histogram was observed.

### Xenograft tumor formation assay

Fourteen virgin TA2 mice aged five to seven weeks were randomly selected and divided into siRNA-NC (n = 7) and siRNA-297 (n = 7) groups. MA-891 cells were treated with siRNA-NC and siRNA-297, as described for transient siRNA transfection. The tumor cells were then resuspended in serum-free RPMI-1640 medium (~1 × 10^6^ cells/100 µL) and injected subcutaneously into the right flank of the mice in the siRNA-NC and siRNA-297 groups (~1 × 10^6^ cells/mouse). From day 6, tumor size was measured and recorded every three days, and the mice were treated with siRNA-NC and siRNA-297 after each recording. All mice were sacrificed on day 21, and the tumors were removed, measured, and photographed. For western blot analysis, protein samples were extracted from fresh tumor tissue, and the rest of the tumor tissue was frozen at -80°C.

### Immunocytochemistry and immunohistochemistry staining

For immunocytochemistry staining, cells were cultured in six-well plates covered with sterilized coverslips and fixed with cold absolute methanol for 30 minutes two days after siRNA transfection. For immunohistochemistry staining, sections were deparaffinized with xylene and sequentially washed with graded ethanol and water. Antigen repair was performed using citrate buffer solution (Zhongshan Inc., China) at 100°C for 10 minutes. The slides were incubated sequentially with endogenous peroxidase inhibitor (Zhongshan Inc., China) and goat serum (Zhongshan Inc., China) for 15 minutes at room temperature. The slides were incubated with primary antibodies (**Supplementary Table 1**) overnight at 4°C. The following day, the samples were incubated sequentially with biotin-labeled goat anti-mouse or rabbit immunoglobulin (Ig) G (Zhongshan Inc., China) and horseradish peroxidase-labeled streptavidin working solution (Zhongshan Inc., China) for 30 and 15 minutes, respectively. Finally, the samples were stained with diaminobenzidine and counterstained with hematoxylin.

### Scratch wound healing assay

A scratch wound healing assay was performed to evaluate the migration ability of siRNA-NC and siRNA-297 cells. Cells were plated in six-well plates and cultured at 37°C with 5% CO_2_. After serum starvation for 12 h, wounds were made at the bottom with sterile pipette tips. The detached cells were washed in sterile PBS before being incubated in a serum-free medium. Scratched areas were recorded at 0, 12, and 24 hours after scratching. ImageJ software was used to compare the scratched areas.

### Migration and invasion assay

The migration and invasion abilities of cells were assessed using a transwell assay. The siRNA-NC and siRNA-297 groups’ cells were diluted to 2.5 × 10^5^/mL before being inoculated into the inserts containing 200 μL of the serum-free medium, while the bottom chamber received 600 μL of medium containing 20% FBS. Then the plates were incubated in a humidified incubator (37°C, 5% CO_2_) for 12 hours. After that, the cell suspension in the inserts was discarded, and the inserts were fixed with absolute methanol for 30 minutes and then stained with 0.1% crystal violet for 30 minutes. The migration ability of the cells was assessed by counting the number of cells/field (× 100). The invasion ability of cells was assessed using a Matrigel-coated transwell assay.

### Co-immunoprecipitation

MA-891 cells were lysed with immunoprecipitation (IP) lysate (Thermo Scientific, USA) in Eppendorf tubes on ice for 30 minutes, followed by centrifugation at 14,000 rpm for 15 minutes at 4°C, and the supernatant was collected. Agarose beads A/G were washed three times with RIPA lysis buffer, and then the protein samples were mixed with agarose beads A/G and incubated on a shaker at 4°C for 30 minutes, followed by centrifugation at 5000 rpm for five minutes at 4°C. The supernatant was then incubated with primary antibodies. Normal rabbit IgG (Beyotime) was used as a control. The following day, two protein samples were mixed separately with agarose beads A/G, washed with RIPA lysate, and incubated at 4°C for two hours. Next, centrifugation was performed at 5000 rpm for five minutes at 4°C, the supernatant was discarded, the pellet was washed three times with RIPA lysate, protein loading buffer was added, it was boiled at 100°C for five minutes, and it was stored at -20°C. Western blot analysis was used to confirm the results of Co-IP.

## Results

### MMTV integrated upstream of the *FGF3* gene of SBCs, and FGF3 was highly expressed in SBCs relative to breast tissues

**Figure 1:**
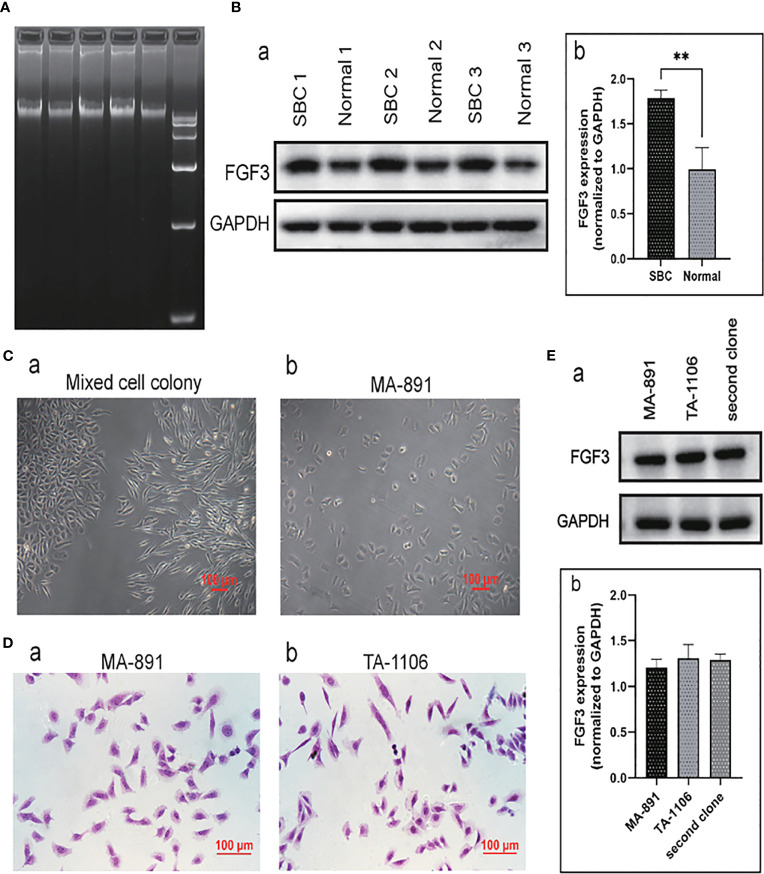
FGF3 expressed in SBC and primary cell culture. **(A)** The agarose gel electrophoresis results of three groups of DNA samples extracted from SBC and DNA markers. **(B)** Western blot analysis of three sets of normal breast tissue and SBC protein samples (a). The expression of FGF3 in SBC and normal breast tissues was quantified and demonstrated by a histogram (b); each bar represents the mean ± standard deviation of three replicates (**P <0.01). **(C)** Morphological characteristics of primary cell mixed cell colony (a) and MA-891 cells (b). **(D)** H&E staining of MA-891 (a) and TA-1106 cells (b). **(E)** Western blot results showed expression levels of FGF3 in MA-891, TA-1106 and cells from a second clone (a). A histogram showing the quantitative results of FGF3 expression in MA-891, TA-1106 cells and cells from a second clone. FGF3, fibroblast growth factor 3; SBC, spontaneous breast cancer; MMTV, mouse mammary tumor virus; TA2, Tientsin albino 2.

To explore the insertion sites of MMTV in the SBC genome, DNA was extracted from SBC tissue. Agarose gel electrophoresis was performed to determine the concentration, purity, and breakage of the DNA samples (**Figure 1A**). The virus integration site analysis of the sequencing result showed that MMTV was inserted 31,091 bp upstream of the *FGF3* gene, located in an intergenic region (**Supplementary Table 3**). Protein samples were extracted from three tumor tissues and their corresponding normal breast tissues, and the expression levels of FGF3 in normal breast tissues and SBC tissues were compared by western blot analysis. The average expression level of FGF3 in SBCs was significantly higher than that in normal breast tissue (**Figure 1B**).

### TA-1106 primary cell culture

MA-891 is a highly metastatic breast cancer cell line derived from TA2 SBC. In addition to MA-891 cells, the primary culture cells TA-1106 were obtained from TA2 SBC. The morphology of primary culture cell clones was compared to that of MA-891 cells (**Figure 1C**). Tumor cell clones with morphologies similar to those of MA-891 were selected for further experiments. Hematoxylin and eosin staining was performed on MA-891 and TA-1106 cells to confirm that the two cell types had similar morphologies (**Figure 1D**). To eliminate potential bias caused by clone selection, we compared the expression levels of FGF3 in MA-891, TA-1106, and a second cell clone through Western blot analysis. The results showed that FGF3 was expressed at similar levels in MA-891, TA-1106 and the second cell clone (**Figure 1E**).

### FGF3 knockdown affected the proliferation and cell cycle of MA-891 and TA-1106 cells

**Figure 2:**
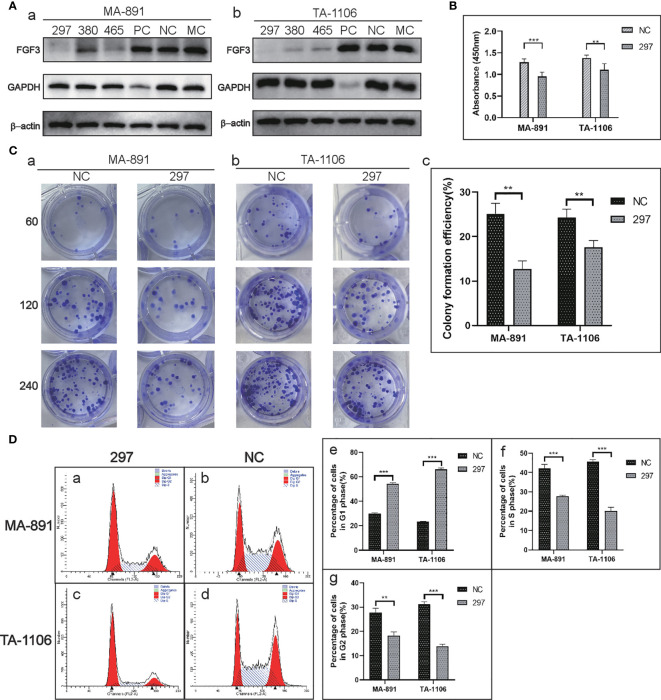
FGF3 knockdown affected the proliferation and cell cycle in MA-891 and TA-1106 cells. **(A)** Comparison of the knockdown effects of siRNA-297, 380, and 465 on FGF3 in MA-891 (a) and TA-1106 (b) cells. **(B)** Absorbance measurements of siRNA-297 cells and siRNA-NC cells at 450 nm by the CCK8 assay. **(C)** A plate colony formation assay showed differences in colony formation ability between siRNA-297 and siRNA-NC from MA-891 (a) and TA-1106 (b), respectively. A histogram showing the quantitative results of the colony-forming ability of MA-891 and TA-1106 cells before and after treatment (c). **(D)** Flow cytometric analyses of cell cycles were performed on siRNA-297 (a) and siRNA-NC (b) from MA-891 and TA-1106 (c, d), respectively. The proportion of cells in G1 phase (e), S phase (f), and G2 phase (g) in each group was also quantitatively compared. Each bar represents the mean ± standard deviation of three independent experiments. Statistically significant differences are indicated as ***P <0.001; **P <0.01. FGF3, fibroblast growth factor 3; CCK8, cell viability cell counting Kit-8; TA2, Tientsin albino 2.

Three candidate siRNA sequences (FGF3 siRNA-297, 380, and 465) were used to knock down the expression; FGF3 siRNA-297 was used for subsequent experiments (**Figure 2A**). The CCK8 assay showed that the absorbance at 450 nm in the siRNA-297 group was significantly lower than that in the siRNA-NC group (**Figure 2B**). The plate clone formation assay demonstrated that the number of clones formed by cells in the siRNA-297 group was significantly less than that of the siRNA-NC group (**Figure 2C**). Subsequently, cell cycle analysis was performed by flow cytometry for the siRNA-297 and siRNA-NC groups, and the results showed that the proportion of cells in the G1 phase in the siRNA-297 group increased, while the proportion of cells in the S and G2 phases decreased, suggesting that FGF3 knockdown can result in G1 cell cycle arrest (**Figure 2D** and **Table 1**).

**Table 1:** Cell cycle analysis by flow cytometry in MA-891 and TA-1106 before and after FGF3 knockdown.

Groups	Dip G1	Dip G2	Dip S
MA-891 siRNA-NC	30.55%	28.69%	40.76%
	29.69%	28.78%	41.53%
	29.92%	25.59%	44.49%
MA-891 siRNA-297	55.16%	17.11%	27.73%
	54.12%	17.54%	28.33%
	52.81%	20.03%	27.15%
TA-1106 siRNA-NC	23.60%	32.04%	44.36%
	22.96%	30.27%	46.78%
TA-1106 siRNA-297	67.30%	14.64%	18.05%
	65.49%	12.98%	21.53%
	65.03%	14.09%	20.89%

### FGF3 knockdown inhibited the migration and invasion of MA-891 and TA-1106 cells

**Figure 3:**
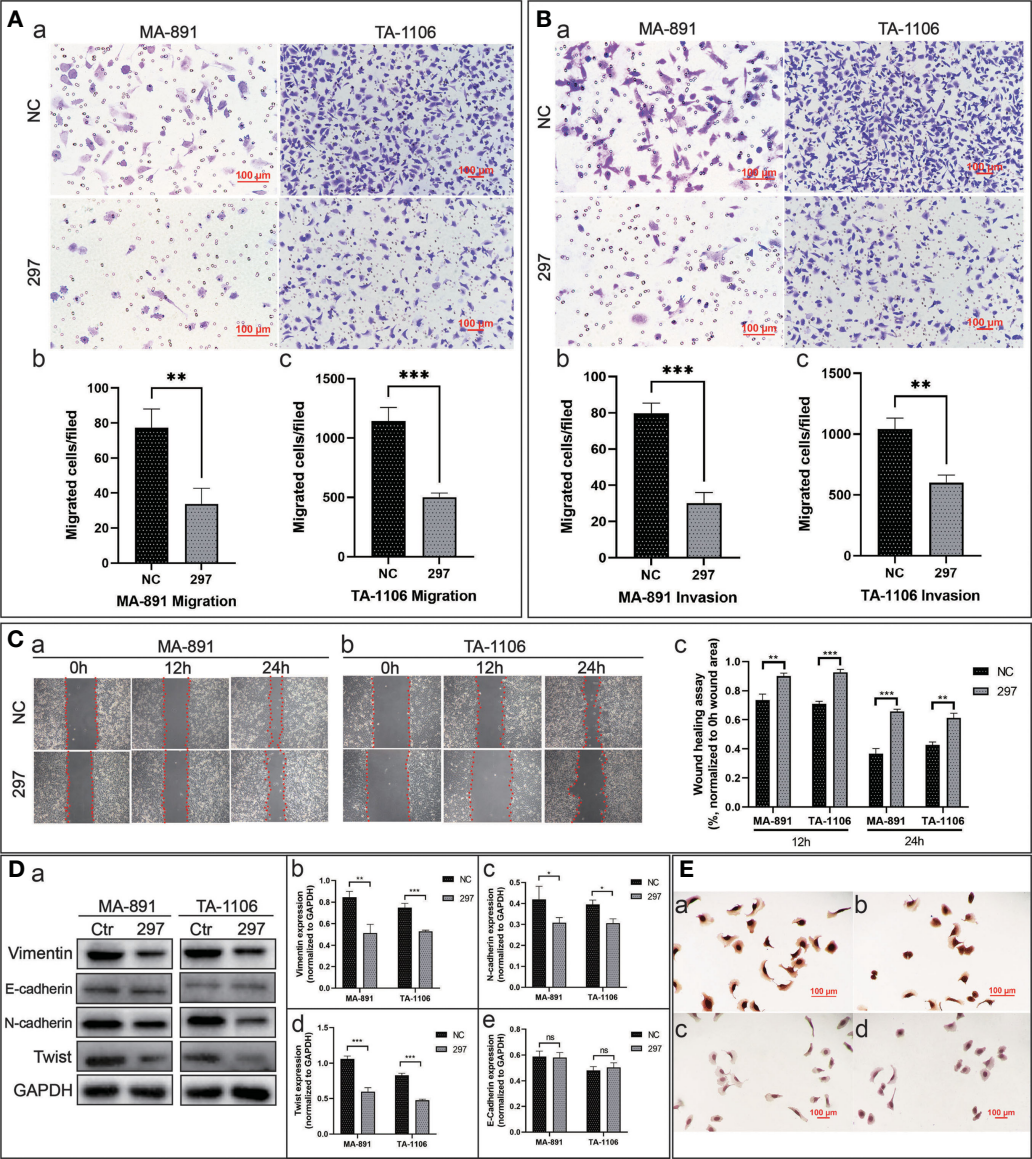
FGF3 knockdown inhibited the migration and invasion abilities of MA-891 and TA-1106 cells. **(A)** Cell migration of siRNA-NC and siRNA-297 cells was assessed by a transwell assay (a). The quantitative analysis results of the transwell migration experiment in MA-891 (b) and TA-1106 (c) after transfection with siRNA-NC or siRNA-297. Each bar represents the mean ± standard deviation (SD) of three independent experiments. **(B)** The cell invasion ability of siRNA-NC and siRNA-297 cells was assessed by a matrigel-coated transwell assay (a). The quantitative analysis results of the transwell invasion experiment in MA-891 (b) and TA-1106 (c) after transfection with siRNA-NC or siRNA-297. **(C)** The scratch wound healing assay was used to assess the cell migration capacity of MA-891 **(A)** and TA-1106 cells **(B)** transfected with siRNA-NC or siRNA-297. A histogram showing the quantitative comparison of scratch wound healing assay results (c). **(D)** Comparison of expression levels of EMT markers between siRNA-297 and siRNA-NC cells by western blot analysis (a). Histograms showing the quantitative results of vimentin (b), N-cadherin (c), Twist (d), and E-cadherin (e) expression in MA-891 and TA-1106 cells transfected with siRNA-NC or siRNA-297. **(E)** Expression levels of N-cadherin in siRNA-NC-transfected MA-891 (a) and TA-1106 cells (b) were detected by ICC, as were the results of siRNA-297-transfected MA-891 (c) and TA-1106 cells (d). Statistically significant differences are indicated as ***P <0.001; **P <0.01; *P v0.05; and ns = not significant. FGF3, fibroblast growth factor 3; EMT, epithelial-mesenchymal transition; TA2, Tientsin albino; ICC, immunocytochemistry.

To explore the effect of FGF3 on the migration ability of tumor cells, a transwell assay was performed. The results showed that more siRNA-NC cells crossed the transwell chamber membrane than siRNA-297 cells (**Figure 3A**). Transwell assays were conducted using Matrigel-coated chambers to compare the invasive ability of cells from the two groups. The results showed that fewer siRNA-297 cells crossed the chamber membrane than the siRNA-NC cells (**Figure 3B**). In the scratch wound healing assay, images of the scratch width for each group of cells were collected and compared at 0, 12, and 24 hours under the same field of view. The results showed that siRNA-297 cells migrated towards the scratch at a slower rate than siRNA-NC cells (**Figure 3C**). Epithelial-mesenchymal transition (EMT)-related proteins were assessed by western blot analysis, the results showed that compared to siRNA-NC cells, vimentin, N-cadherin, and Twist were significantly downregulated in siRNA-297 cells, while there was no significant difference in the expression level of E-cadherin, immunocytochemical staining also confirmed the downregulation of N-cadherin in siRNA-297 cells (**Figures 3D, E**).

### FGF3 knockdown inhibited STAT3 and Akt-related pathways and suppressed tumor growth

**Figure 4:**
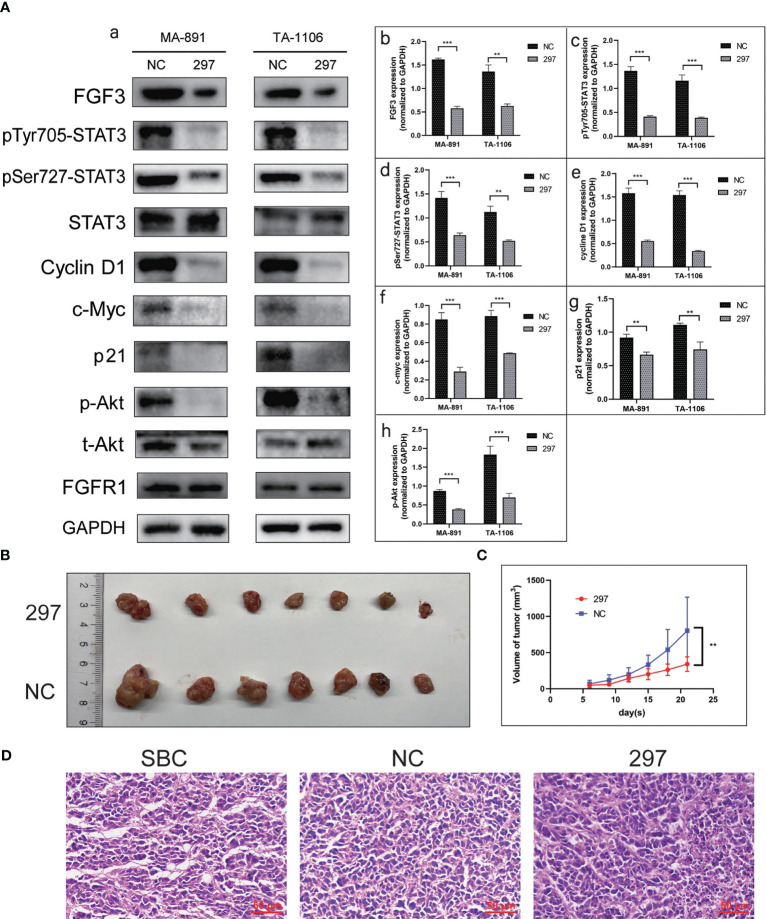
FGF3 knockdown suppressed STAT3 and Akt-related pathways as well as the growth of xenografts. **(A)** Western blot results showed expression levels of FGF3, p-STAT3^Tyr705^, p-STAT3^Ser727^, STAT3, Cyclin D1, c-Myc, p21, p-Akt, t-Akt, and FGFR1 in siRNA-297 and siRNA-NC cells (a). Histograms of quantitative differences in expression of FGF3 (b), p-STAT3^Tyr705^ (c), p-STAT3^Ser727^ (d), Cyclin D1 (e), c-Myc (f), p21 (g), and p-Akt (h) in siRNA-NC and siRNA-297-transfected MA-891 and TA-1106 cells. Each bar represents the mean ± standard deviation of three independent experiments. Statistically significant differences are indicated as: ***P <0.001; **P <0.01. **(B)** Comparison of siRNA-297 and siRNA-NC cell xenografts. **(C)** Tumor growth curves of TA2 mouse xenografts, MA-891 siRNA-297 cells, and siRNA-NC cells are compared. Statistically significant differences are indicated as **P <0.01. **(D)** H&E staining was used to compare the morphology of SBC, siRNA-NC and siRNA-297-transfected MA-891 cell xenografts. SBC, spontaneous breast cancer; t-Akt, total Akt; p-Akt, phosphorylated-Akt; FGF3, fibroblast growth factor 3; H&E, hematoxylin and eosin; TA2, Tientsin albino 2.

**Figure 5:**
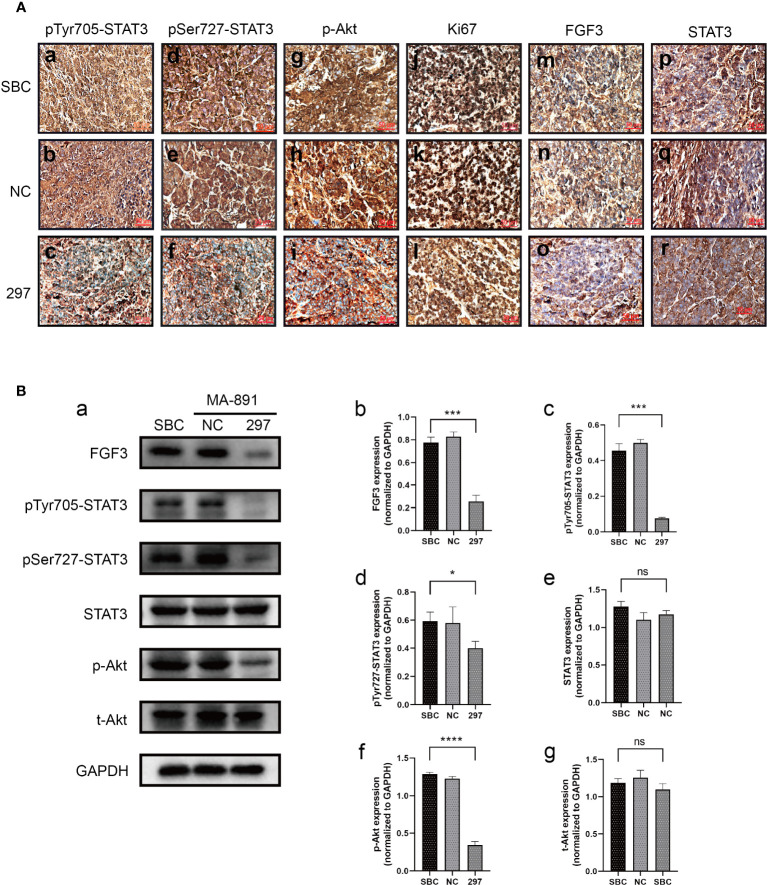
FGF3 knockdown suppressed STAT3 and Akt-related pathways in xenografts. **(A)** Comparison of expression levels of p-STAT3^Tyr705^ (a-c), p-STAT3^Ser727^ (d-f), p-Akt (g-i), Ki67 (j-l), FGF3 (m-o), and STAT3 (p-r) among SBC, siRNA-NC and siRNA-297-transfected MA-891 cell xenografts by immunohistochemistry. **(B)** Western blot analysis of the expression of proteins including FGF3, p-STAT3^Tyr705^, p-STAT3^Ser727^, STAT3, p-Akt, and t-Akt in SBC and xenografts with siRNA-NC and siRNA-297 treatments (a). Histograms of quantitative differences in expression of FGF3 (b), p-STAT3^Tyr705^ (c), p-STAT3^Ser727^ (d), STAT3 (e), p-Akt (f), and t-Akt (g) in SBC, siRNA-NC and siRNA-297-transfected MA-891 xenografts. Statistically significant differences are indicated as ****P <0.0001;***P <0.001; *P <0.05; and ns, not significant. SBC, spontaneous breast cancer; t-Akt, total Akt; p-Akt, phosphorylated-Akt; FGF3, fibroblast growth factor 3; FGFR1, fibroblast growth factor receptor 1; TA2, Tientsin albino 2.

To determine the underlying mechanism of the FGF3-induced malignant phenotype, we explored the relationship between FGF3, STAT3, and Akt. FGF3 knockdown significantly inhibited the expression of p-STAT3^Tyr705^, p-STAT3^Ser727^, p-Akt, p21, Cyclin D1, and c-Myc (**Figure 4A**). MA-891 cells from the siRNA-297 and siRNA-NC groups were injected subcutaneously into the right flank of the TA2 mice. The size of the xenografts treated with siRNA-NC and siRNA-297 was measured. All mice were sacrificed 21 days after injection, and the tumor size and expression of protein were compared between the two groups. The results showed that the tumor size of MA-891 cells in the siRNA-297 group was significantly smaller than that in the siRNA-NC group (**Figures 4B, C**). Hematoxylin and eosin staining was used to compare the morphology of the SBC, siRNA-NC xenografts, and siRNA-297 xenografts (**Figure 4D**). Compared to SBC and siRNA-NC xenografts, the expression levels of p-STAT3^Tyr705^, p-STAT3^Ser727^, p-Akt, Ki67 and FGF3 were decreased in siRNA-297 xenografts, while there was no significant difference in expression level of STAT3 (**Figures 5A, B**).

### FGF3 bound to FGFR1 to activate STAT3 and Akt

**Figure 6:**
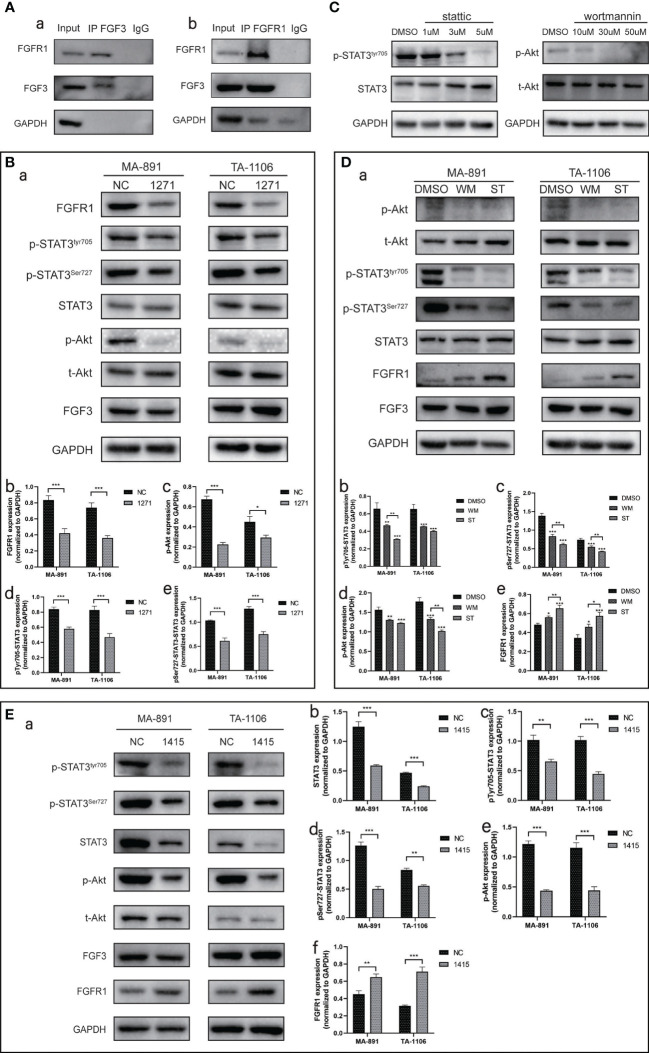
The expression of FGF3-related proteins. **(A)** Co-IP experiments were performed on MA-891 cells using either FGF3 (a) or FGFR1 (b) as bait to examine the binding of FGFR1 and FGF3, respectively. **(B)** The expression levels of FGFR1, p-STAT3^Tyr705^, p-STAT3^Ser727^, STAT3, p-Akt, t-Akt, and FGF3 in siRNA-NC and siRNA-1271 cells were compared after FGFR1 was knocked down (a). Histograms of quantitative differences in the expression of FGFR1 (b), p-Akt (c), p-STAT3^Tyr705^ (d), and p-STAT3^Ser727^ (e) in siRNA-NC and siRNA-297-transfected MA-891 and TA-1106 cells. Each bar represents the mean ± standard deviation of three independent experiments. Statistically significant differences are indicated as: ***P < 0.001; **P <0.01; *P <0.05. **(C)** Expression levels of p-STAT3^Tyr705^ and p-Akt under the treatment of different concentration gradients of ST and WM, respectively. **(D)** Effects of WM and ST treatment on p-Akt, t-Akt, p-STAT3^Tyr705^, p-STAT3^Ser727^, STAT3, FGFR1, and FGF3 levels (a). Histograms of quantitative differences in expression of p-STAT3^Tyr705^ (b), p-STAT3^Ser727^ (c), p-Akt (d), and FGFR1 (e) in DMSO, WM, and ST-treated MA-891 and TA-1106 cells. **(E)** STAT3 was knocked down by siRNA-1415, and the expression levels of p-STAT3^Tyr705^, p-STAT3^Ser727^, STAT3, p-Akt, t-Akt, FGF3, and FGFR1 were compared between siRNA-NC and siRNA-1415 cells (a). Histograms of quantitative differences in expression of STAT3 (b), p-STAT3^Tyr705^ (c), p-STAT3^Ser727^ (d), p-Akt (e), and FGFR1 (f) in siRNA-NC and siRNA-1415-transfected MA-891 and TA-1106 cells. Co-IP, co-immunoprecipitation; t-Akt, total Akt; p-Akt, phosphorylated-Akt; DMSO, dimethyl sulfoxide; WM, *Wortmannin*; ST, *Stattic*; FGF3, fibroblast growth factor 3; FGFR1, fibroblast growth factor receptor 1; TA2, Tientsin albino 2.

Bioinformatic analysis was used to analyze the functional residues of FGF3 and FGFR1 using LigPlot^+^ version 2.2.4. In hydrogen bonding interactions, many residues contribute to the construction, such as Asn-77 of FGFR1 and Glu-123 of FGF3. Some of the remaining residues, such as Tyr-152 of FGFR1 and Arg-120 of FGF3, were used to form hydrophobic interactions. All contributing residues are shown as 2D interactions. In contrast, we got the exact binding energy and affinity (Kd) scores from Prodigy: -10.5 kcal/mol and 2.0 e-08 M, respectively. PyMol 2.2.0 was used to visualize the protein-protein docking conformation. FGFR1 was represented as a slate cartoon, FGF3 was shown as a cyan cartoon, and 3D interactions and their binding sites are shown (**Supplementary Figures 1A–1D**). We then directly verified the binding of FGF3 to FGFR1 using Co-IP (**Figure 6A**). FGFR1 was then knocked down with siRNA-1271, and the phosphorylation levels of STAT3 and Akt subsequently decreased (**Figure 6B**). Based on the results of the previous section, we conclude that FGF3 can activate STAT3 and Akt-related signaling pathways by binding to FGFR1.

### Relationship between STAT3 and Akt phosphorylation

To explore the relationship between STAT3 and Akt-related pathways, we inhibited STAT3 and Akt phosphorylation with *Stattic* (ST) and *Wortmannin* (WM), respectively. The levels of p-STAT3^Tyr705^ and p-Akt gradually decreased in response to increasing ST and WM concentrations (**Figure 6C**). However, excessive drug concentrations severely inhibited the growth of MA-891 cells; therefore, we selected a concentration of 3 µM ST and 30 µM WM for subsequent experiments. We found that inhibition of Akt phosphorylation by WM also led to a decreased STAT3 phosphorylation level. Interestingly, inhibition of STAT3 phosphorylation by ST also led to a decreased Akt phosphorylation level. Another interesting finding was that both WM and ST could increase the expression of FGFR1, and the effect of ST was stronger than that of WM (**Figure 6D**). We also knocked down STAT3 with siRNA-1415, and the results showed that the Akt phosphorylation level was decreased and the expression level of FGFR1 was increased (**Figure 6E**). To investigate whether there was increased binding of FGF3 and FGFR1 in siRNA-1415 cells compared to siRNA-NC cells, a Co-IP experiment was conducted. The results revealed that an equivalent amount of FGF3 exhibited enhanced binding with FGFR1 in siRNA-1415 cells (**Supplementary Figure 1E**).

## Discussion

The WGS results of the TA2 SBC sample in this study verified that SBC was caused by MMTV insertion, corroborating previous findings (8–10). The insertion site of MMTV was 31,091 bp upstream of the *FGF3* gene, and it was speculated that the enhancer in the MMTV genome promoted *FGF3* upregulation, which was in accordance with previous studies (23). It is worth mentioning that only one MMTV insertion site was found in the WGS; therefore, we can infer that FGF3 has a strong role in promoting the development of SBC, which also reflects the significance of studying the cancer-promoting mechanism of FGF3. This study showed that the mean expression level of FGF3 was higher in SBCs than in normal breast tissues, suggesting a general association between FGF3 and SBCs. Contrastingly, because TA2 SBC belongs to TNBC (6, 7), there are not many cases of elevated expression of FGF3 in HBC; therefore, it is necessary to explore whether there is a relationship between FGF3 and TNBC in HBC. In this study, WGS was performed on one SBC sample, focusing on the role of FGF3 in the development of SBC. The polymerase chain reaction is currently used in studies aimed at discovering novel insertion sites for MMTV (24, 25).

Cell function assays confirmed that FGF3 plays an important role in tumor cell proliferation, cell cycle, migration, invasion, EMT, and xenograft formation. Therefore, we speculate that for breast cancer with high expression of FGF3, treatment against FGF3 is worth exploring and that FGF3 may be used as a drug target. In this study, we simulated the binding of mouse FGF3 to FGFR1 using molecular docking and verified the binding between the two using Co-IP. Prior to this, only Orn et al. verified that FGF3 could activate FGFR1-1b-induced cell division-related pathways by assaying mitogenic activity using engineered BaF3 cell lines (26). This study provides more direct evidence that FGF3 binds to FGFR1, and engineered cell lines were not used in this study, reflecting the endogenous experimental results.

STAT3 has previously been shown to influence the malignant phenotype of MA-891 cells. Inhibition of STAT3 phosphorylation with small molecule inhibitors Cryptotanshinone (CTS) and ST significantly suppressed the proliferation, migration, and invasion capabilities of MA-891 cells, as well as the growth of MA-891 cell xenografts (8). This study demonstrated that FGF3 knockdown inhibited STAT3 phosphorylation, while FGFR1 knockdown had a similar effect. Therefore, we hypothesized that FGF3 binding to FGFR1, leading to STAT3 phosphorylation, is one of the pathways by which FGF3 exerts its cancer-promoting function. Furthermore, we demonstrated that FGF3/FGFR1 could also lead to Akt phosphorylation. In addition to binding to FGFR1, FGF3 can also bind to FGFR2 (26, 27). Besides, our previous study has shown that FGFR2 was highly expressed in SBC compared to normal breast tissue (8). Prolonged FGFR1 knockdown may trigger compensatory mechanisms in tumor cells. Transient siRNA transfection was used in this study, and sample proteins were collected for western blot analysis two days after siRNA transfection, thus ensuring the effect of siFGFR1. We also investigated the association between the Akt and STAT3 pathways. Several studies have investigated the Akt/STAT3 pathway (28–30). Xu et al. showed that STAT3 bound to the Akt1 promoter and promoted the expression of Akt1 (31). However, in this study, we found that STAT3 only affected Akt phosphorylation and did not affect the expression of Akt. At the same time, Akt also affected STAT3 phosphorylation without affecting its expression. Based on these results, inhibiting STAT3 and Akt in breast cancer with higher expression levels of FGF3 could be used as a potential measure to treat breast cancer or as a complementary means for FGFR-targeted therapy.

FGFR signaling has been shown to be associated with various malignant phenotypes, such as cell proliferation, migration, invasion, and angiogenesis, making FGFR-targeted therapies a viable option for cancer patients (32, 33). FGFR-targeted drugs can be classified into different types, including tyrosine kinase inhibitors, fibroblast growth factor ligand traps, and monoclonal antibodies. These targeted drugs have been developed for the clinical treatment of different types of tumors, including breast cancer (34). Studies have indicated that the efficacy of pan-FGFR inhibitors, such as Rogatimib, in tumor treatment is closely related to the expression levels of FGFR genes. They exhibit better therapeutic effects on cell lines and xenografts with high FGFR expression levels (35). We found FGFR1 upregulation by WM or ST treatment and it should be further verified whether STAT3 or Akt inhibitors have an upregulation effect on other FGFRs and, further, whether there is a similar upregulation effect in HBC or other tumors. Investigating this issue may help address resistance to FGFR-targeted therapies or determine whether tumors that fail to respond to FGFR-targeted therapies because of low expression levels of FGFR can be converted to sensitivity to FGFR-targeted therapies by upregulating expression of FGFR through WM or ST treatment, all of which deserve further investigation.

In this study, we did not compare the roles of different subtypes of FGFRs in the action of FGF3, so it remains unclear which subtype of FGFR plays a major role in mediating the effects of FGF3. We demonstrated through experiments that there is a reciprocal activation between STAT3 and Akt, but the specific mechanisms underlying this process were not deeply investigated. Additionally, it would be valuable to collect clinical samples and compare the expression levels of FGF3 in various molecular subtypes of breast cancer and normal breast tissue/benign breast lesions to explore the proportion of FGF3 overexpression in HBC. We compared the expression levels of FGF3 in HBC and normal breast tissue using data from the TCGA database and GTEx database. The results showed that although FGF3 is not universally overexpressed in HBC, its expression level in HBC is significantly higher than that in normal breast tissue (**Supplementary Figure 1F**).

**Figure 7:**
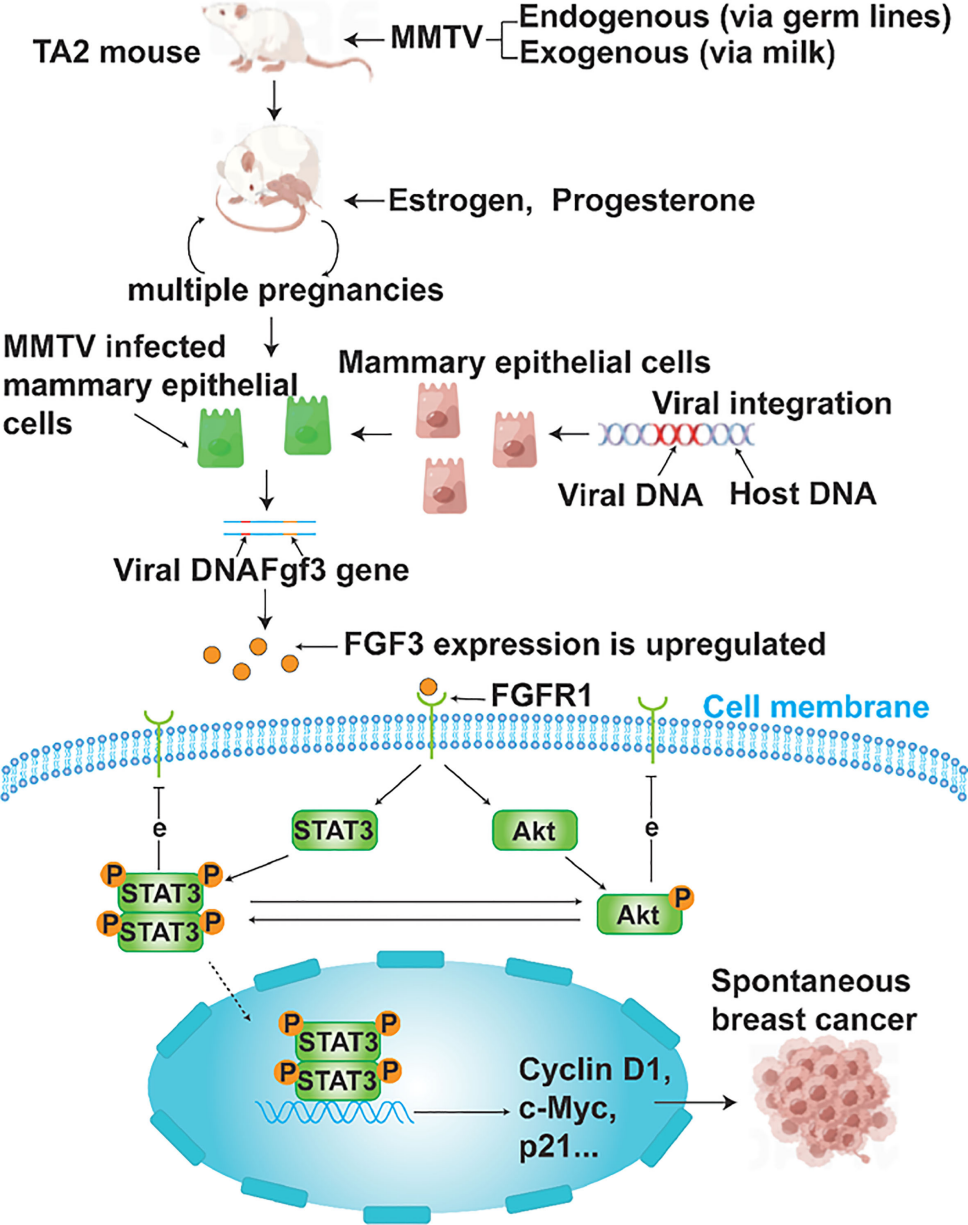
A schematic representation of MMTV causing SBC in TA2 mice. TA2 mice transmit MMTV through breast milk or genetic material. TA2 mice with MMTV integrated into their DNA become pregnant several times and have active mammary epithelial cell division in response to estrogen and progesterone. Frequent DNA replication processes lead to the amplification of MMTV as well, and newly produced viral particles can infect mammary epithelial cells. Enhancer elements in viral sequences cause oncogene overexpression when the virus integrates near an oncogene. In this study, FGF3 is overexpressed. FGF3 binds to FGFR1 and regulates STAT3 and Akt phosphorylation as well as the expression of cyclin D1, c-myc, and p21, which contributes to the development of SBC. Phosphorylated STAT3 promotes Akt phosphorylation and *vice versa.* Both phosphorylated STAT3 and Akt inhibit the expression of FGFR1.

At the end of this article, a diagram was utilized to depict the process of MMTV-induced SBC in TA2 mice and summarize the key findings of this study (**Figure 7**).

## Conclusion

Our results indicate that FGF3 is highly expressed in the SBCs of TA2 mice and is associated with tumor cell proliferation, cell cycle, migration, EMT, invasion, and tumorigenesis in xenografts. FGF3 binds to FGFR1 to regulate STAT3 phosphorylation and expression of cyclin D1, c-Myc, and p21, thereby inducing SBC formation. Additionally, FGF3 can regulate Akt phosphorylation. Inhibition of STAT3 phosphorylation led to decreased phosphorylation levels of Akt, and inhibition of Akt phosphorylation also resulted in decreased phosphorylation levels of STAT3. Furthermore, inhibition of Akt or STAT3 phosphorylation caused FGFR1 upregulation. Next, the binding sites of FGF3 and FGFR1 were experimentally validated. The homologous sequences of MMTV in HBC may explain the tumorigenic mechanism of breast cancer.

## Data availability statement

The datasets presented in this study can be found in online repositories. The names of the repository/repositories and accession number(s) can be found in the article/**Supplementary Material**.

## Ethics statement

The animal study was reviewed and approved by Tianjin Union Medical Center.

## Author contributions

SiZ and ShZ designed the paper, contributed to manuscript writing and approved the manuscript before submission. LC, XZ and GL collected literatures and approved the manuscript before submission. SC and MZ gave constructive comments on the manuscript, and approved the manuscript before submission.
