# Supplementary material for: Fibroblast growth factor 3 promotes spontaneous mammary tumorigenesis in Tientsin albino 2 mice via the FGF3/FGFR1/STAT3 pathway
Source: Front Oncol. 2023 Jul 11;13:1161410. doi: 10.3389/fonc.2023.1161410 (PMC10367089; doi:10.3389/fonc.2023.1161410)
Supplement: Supplementary file 1 [file DataSheet_1.docx]

**Supplementary Figure 1 Legends**


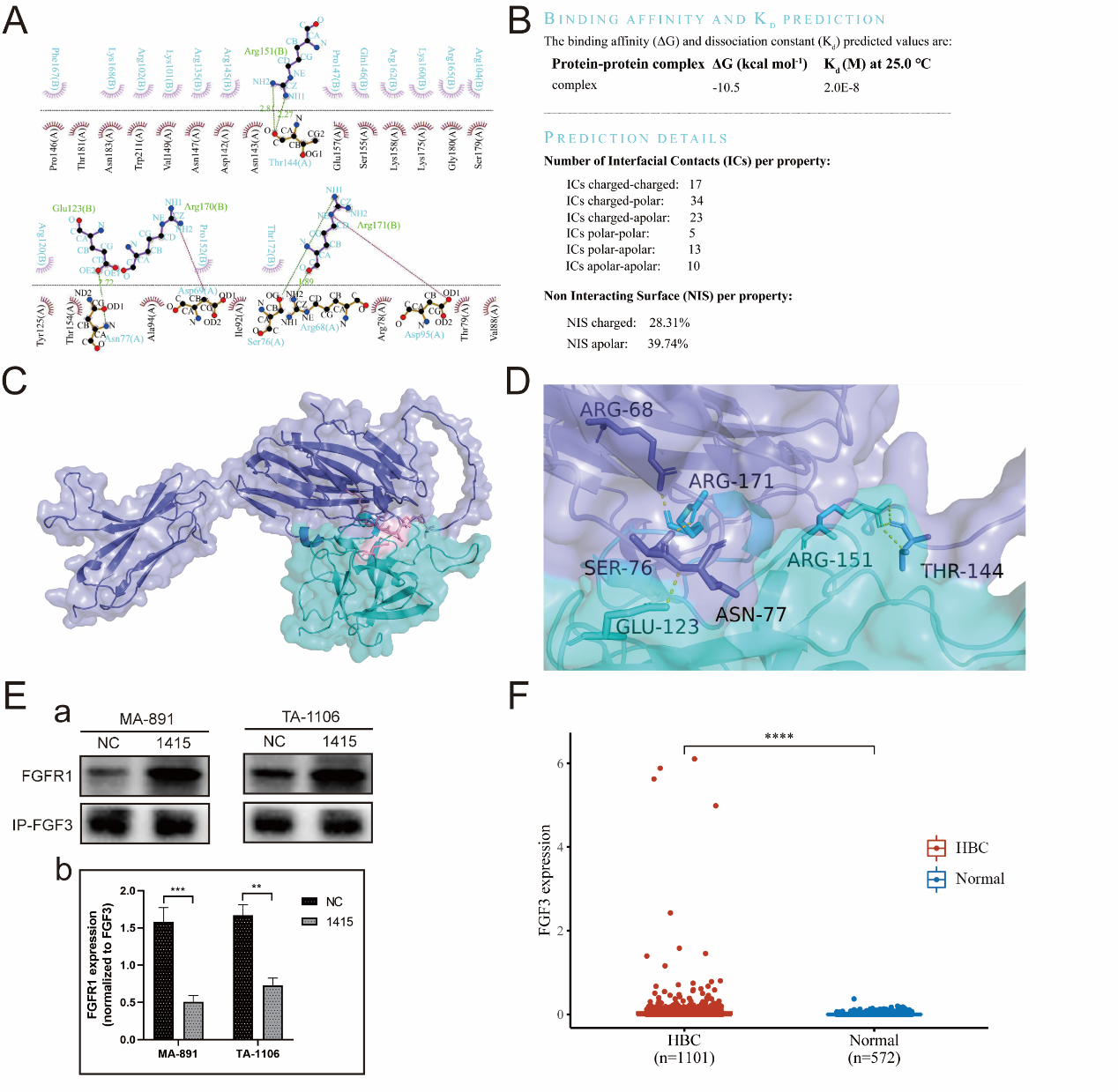


(A) 2D presentation of residues contributing to FGFR1 binding to FGF3, where FGFR1 was labeled as A and FGF3 as B. (B) Prodigy predicted the binding energy and dissociation constant between FGF3 and FGFR1 to be -10.5 kcal/mol and 2.0 e-08 M, respectively. (C) PyMol presented a 3D model of FGFR1–FGF3 interaction, in which FGFR1 was represented as a slate cartoon, FGF3 was shown as a cyan cartoon, and their binding sites were shown as pink sticks. (D) 3D presentation of residues playing roles in the binding of FGF3 to FGFR1. FGFR1 was represented as a slate cartoon, and FGF3 was shown as a cyan cartoon. (E) Comparison of quantity of FGFR1 between siRNA-NC and siRNA-1415 cells after Co-IP experiment using FGF3 as bait by western blot analysis (a). The histogram showed the quantitative results of FGFR1 expression in MA-891 and TA-1106 cells transfected with siRNA-NC or siRNA-1415 after setting FGF3 to an equivalent level (b). Each bar represents the mean ± standard deviation of three independent experiments. Statistically significant differences are indicated as: *****P* <0.0001; ****P* <0.001; ***P* <0.01; **P* <0.05. (F) The expression of FGF3 in 1,101 cases of HBC from the TCGA database was compared with the expression in 572 cases of normal breast tissue from the TCGA database plus the GTEx database using the Wilcoxon test.

Co-IP, co-immunoprecipitation; HBC, human breast cancer; TCGA, The Cancer Genome Atlas; GTEx, The Genotype-Tissue Expression.

**Supplementary Table 1.** Detail information of antibodies used in this study.

| Antibodies | Companies | Dilution | | | |
| --- | --- | --- | --- | --- | --- |
|  |  | WB | ICC | IHC | CoIP |
| p-STAT3^Tyr705^ | Affinity | 1:1000 |  | 1:100 |  |
| p-STAT3^Ser727^ | Affinity | 1:500 |  | 1:100 |  |
| FGFR1 | Santa Cruz | 1:200 |  |  | 1:50 |
| FGF3 | Santa Cruz | 1:200 |  |  | 1:50 |
| STAT3 | Wanleibio | 1:500 |  |  |  |
| p-Akt | Santa Cruz | 1:200 |  | 1:200 |  |
| t-Akt | Santa Cruz | 1:200 |  |  |  |
| p21 | Affinity | 1:500 |  |  |  |
| c-Myc | Affinity | 1:500 |  |  |  |
| Cyclin D1 | Affinity | 1:1000 |  |  |  |
| Ki67 | Abcam |  |  | 1:500 |  |
| E-cadherin | Affinity | 1:1000 |  |  |  |
| N-cadherin | Affinity | 1:1000 | 1:100 |  |  |
| Vimentin | Wanleibio | 1:500 |  |  |  |
| Twist | Wanleibio | 1:500 |  |  |  |
| GAPDH | Affinity | 1:3000 |  |  |  |
| β-actin | Affinity | 1:3000 |  |  |  |

**Supplementary Table 2.** Detail information of siRNA sequences.

| **siRNA** | **Sense (5’-3’)** | **Antisense (5’-3’)** |
| --- | --- | --- |
| FGF3i-297 | CCUUGAGAACAGCGCCUAUTT | AUAGGCGCUGUUCUCAAGGTT |
| FGF3i-380 | GGUACCUGGCCAUGAACAATT | UUGUUCAUGGCCAGGUACCTT |
| FGF3i-465 | GCUGGGCUACAAUACAUAUTT | AUAUGUAUUGUAGCCCAGCTT |
| FGF3i-PC | CACUCAAGAUUGUCAGCAATT | UUGCUGACAAUCUUGAGUGAG |
| FGF3i-NC | UUCUCCGAACGUGUCACGUTT | ACGUGACACGUUCGGAGAATT |
| FGFR1i-1271 | CCAAGACGGUGAAGUUCAATT | UUGAACUUCACCGUCUUGGTT |
| STAT3i-1415 | GGGUCUCGGAAAUUUAACATT | UGUUAAAUUUCCGAGACCCTT |

PC: GAPDH positive control; NC: Negative control; FGF3i: FGF3 knockdown; FGFR1i: FGFR1 knockdown; STAT3i: STAT3 knockdown

**Supplementary Table 3.** The integration site for MMTV inserting SBC.

| Chr | Start | End | Func | Gene | GeneDetail |
| --- | --- | --- | --- | --- | --- |
| chr7 | 1.45E+08 | 1.45E+08 | intergenic | NM_178642 (*Ano1*), NM_008007 (*Fgf3*) | dist=68929;dist=31091 |
| chr7 | 54234123 | 54234123 | intergenic | NR_105746(*Mir6238*), NM_178705 (*Luzp2*) | dist=342234;dist=601122 |

MMTV, mouse mammary tumor virus; SBC, spontaneous breast cancer
